# 第三代EGFR-TKIs在老年非小细胞肺癌中的研究现状及进展

**DOI:** 10.3779/j.issn.1009-3419.2025.101.09

**Published:** 2025-05-20

**Authors:** Xue CHEN, Yijia SUN, Lihong ZHANG, Bo JIANG

**Affiliations:** ^1^650500 昆明，昆明医科大学; ^1^Kunming Medical University, Kunming 650500, China; ^2^650118 昆明，昆明医科大学第三附属医院老年肿瘤科; ^2^Department of Geriatric Oncology, the Third Affiliated Hospital of Kunming Medical University, Kunming 650118, China

**Keywords:** 肺肿瘤, 表皮生长因子受体-酪氨酸激酶抑制剂, 研究现状, 研究进展, Lung neoplasms, Epidermal growth factor receptor-tyrosine kinase inhibitors, Research status, Research progress

## Abstract

对于表皮生长因子受体（epidermal growth factor receptor, *EGFR*）敏感突变的晚期非小细胞肺癌（non-small cell lung cancer, NSCLC）患者，指南优先推荐第三代EGFR-酪氨酸激酶抑制剂（EGFR-tyrosine kinase inhibitors, EGFR-TKIs），其客观缓解率（objective response rate, ORR）更高、无进展生存期（progression-free survival, PFS）更长且生活质量更优。然而，老年患者因临床试验入组比例较低，现有证据难以完全指导该人群的临床实践。本文通过整合前瞻性及回顾性研究中的亚组分析或预先指定的研究目的，探讨第三代EGFR-TKIs单药或联合治疗在老年NSCLC人群中的疗效与安全性差异。结果显示，第三代EGFR-TKIs在老年患者中疗效与年轻患者中相当且安全性良好。联合方案虽能延长生存期，但毒性叠加，需权衡风险。

## Abstract (en)

For patients with advanced non-small cell lung cancer (NSCLC) harboring sensitive epidermal growth factor receptor (*EGFR*) mutations, guidelines prioritize the use of third-generation EGFR-tyrosine kinase inhibitors (EGFR-TKIs), which offer higher objective response rate (ORR), longer progression-free survival (PFS), and better quality of life. However, due to the low proportion of elderly patients enrolled in clinical trials, the existing evidence is insufficient to fully guide clinical practice. This review examines the efficacy and safety differences of third-generation EGFR-TKIs as monotherapy or in combination in the elderly NSCLC by integrating subgroup analyses or pre-specified research objectives from prospective and retrospective studies. The results show that third-generation EGFR-TKIs have comparable efficacy in elderly patients to younger populations and are well-tolerated. Although combination therapies may extend survival time, the associated increased toxicity necessitates careful risk-benefit assessment.

肺癌是全球最常见癌症及癌症相关死亡首要原因，占全球新发癌症的12.4%和癌症死亡的18.7%^[1]^，其中非小细胞肺癌（non-small cell lung cancer, NSCLC）占比80%-85%。全球疾病负担（global burden of disease, GBD）数据^[2]^显示2019年各年龄组中肺癌发病人数呈近似正态分布，65-69岁年龄组人群肺癌新发病例数最多，70-74岁年龄组人群肺癌死亡人数最多，凸显其老年性疾病特征。随着老龄化加剧，老年肺癌患者比例将持续上升，但现有临床试验对老年人群研究不足^[3]^，且该群体普遍存在生理功能衰退、并发症多、社会支持薄弱等问题^[4]^，使得临床医生在制定老年肺癌患者的治疗方案时面临更大挑战。

超过50%的肺癌患者确诊时已处于晚期，失去手术机会。靶向治疗因其疗效显著且毒性可控，成为晚期NSCLC重要治疗手段，特别是表皮生长因子受体-酪氨酸激酶抑制剂（epidermal growth factor receptor-tyrosine kinase inhibitors, EGFR-TKIs）。目前临床应用的EGFR-TKIs包括：第一代吉非替尼、厄洛替尼，第二代阿法替尼和达克替尼及第三代奥希替尼、阿美替尼和伏美替尼等。这些药物显著改善了*EGFR*敏感突变患者的生存预后。

然而，*EGFR*突变NSCLC的肿瘤生物学行为是否存在年龄差异，以及EGFR-TKIs在不同年龄人群中的疗效异同仍不明确。本文通过整合前瞻性与回顾性研究数据，重点分析第三代EGFR-TKIs单药或联合方案在老年患者中的疗效及安全性差异，旨在为老年患者精准治疗提供更多依据。

## 1 第一代及第二代EGFR-TKIs在老年NSCLC中的研究现状

Roviello等^[5]^的*meta*分析表明，与年轻患者相比，EGFR-TKIs在延长老年患者无进展生存期（progression-free survival, PFS）方面更有效，是*EGFR*突变老年患者的有效治疗手段。韩等^[6]^在一项关于老年与青年NSCLC患者对EGFR-TKIs治疗反应的系统评价中表明，在携带*EGFR*突变的NSCLC患者中，包括青年（<45岁）和老年患者（≥65岁），靶向治疗对延缓老年晚期NSCLC患者的疾病进展尤其有效，青年NSCLC患者接受EGFR-TKIs治疗的获益程度低于老年患者。陈等^[7]^的系统评价也显示，相比年龄<65岁患者，EGFR-TKIs可以进一步降低*EGFR*敏感突变型老年晚期NSCLC患者的无疾病进展风险，可能与老年患者机体整体代谢速度较慢导致肿瘤生长速度受影响有关。多项系统评价与*meta*分析^[5-7]^中指出，EGFR-TKIs在老年患者中疗效显著，尤其在延长PFS方面优于年轻患者。然而，上述研究主要针对第一代和第二代EGFR-TKIs，第三代EGFR-TKIs在老年患者中的研究较少。根据指南推荐^[8]^，对于*EGFR*敏感突变的晚期NSCLC患者，优先选择第三代EGFR-TKIs，但其在老年患者中的疗效和安全性仍需进一步研究。

## 2 第三代EGFR-TKIs在老年NSCLC中的研究进展

### 2.1 第三代EGFR-TKIs单药治疗在老年NSCLC中的研究进展

对于*EGFR*敏感突变的晚期NSCLC患者，指南推荐优先选择第三代EGFR-TKIs。目前在中国上市的第三代EGFR-TKIs有奥希替尼、阿美替尼、伏美替尼等。表1^[9-14]^及表2^[15-18]^汇总了目前I-III期临床试验中第三代EGFR-TKIs在老年NSCLC患者中的研究结果。

**表 1:** 第三代EGFR-TKIs单药治疗NSCLC的III期临床试验

Study	Sample size	Line of treatment	Experiment arm	Control arm	PFS by age HR (95%CI)
FLAURA^[9]^	*N*=556 <65 years: *n*=298 ≥65 years: *n*=258	First line	Osimertinib	Gefitinib or Erlotinib	<65 years: 0.44 (0.33-0.58); ≥65 years: 0.49 (0.35-0.67)
AURA3^[10]^	*N*=419 <65 years: *n*=242 ≥65 years: *n*=177	Second line	Osimertinib	Platinum-Pemetrexed	<65 years: 0.38 (0.28-0.54); ≥65 years: 0.34 (0.23-0.50)
APOLLO^[11]^	*N*=244 <65 years: *n*=153 ≥65 years: *n*=91	Second line	Aumolertinib	-	<65 years: 0.38 (0.28-0.54); ≥65 years: 0.34 (0.23-0.50)
AENEAS^[12]^	*N*=429 <65 years: *n*=294 ≥65 years: *n*=135	First line	Aumolertinib	Gefitinib	<65 years: 0.44 (0.33-0.59); ≥65 years: 0.54 (0.34-0.88)
FURLONG^[13]^	*N*=357 <65 years: *n*=232 ≥65 years: *n*=125	First line	Furmonertinib	Gefitinib	<65 years: 0.36 (0.26-0.51); ≥65 years: 0.68 (0.43-1.09)
NCT04206072^[14]^	*N*=362 <65 years: *n*=260 ≥65 years: *n*=102	First line	Befotertinib	Icotinib	<65 years: 0.42 (0.29-0.60); ≥65 years: 0.79 (0.43-1.42)

NSCLC: non-small cell lung cancer; EGFR-TKIs：epidermal growth factor receptor-tyrosine kinase inhibitors; PFS: progression-free survival; HR: hazard ratio.

**表 2:** 第三代EGFR-TKIs单药治疗NSCLC的I或II期临床试验

Study	Sample size	Line of treatment	Treament arm	ORR (95%CI)
NCT03861156^[15]^	*N*=290 <65 years: *n*=172 ≥65 years: *n*=118	Second line	Befotertinib	<65 years: 69.8% (62.3%-76.5%); ≥65 years: 64.4% (55.1%-73.0%)
NCT03812809^[16]^	*N*=226 <65 years: *n*=163 ≥65 years: *n*=63	Second line	Rezivertinib	<65 years: 64.4% (56.7%-71.7%); ≥65 years: 65.1% (52.0%-76.7%)
NCT03386955^[17]^	*N*=43 <50 years: *n*=6 50-65 years: *n*=20 ≥65 years: *n*=17	First line	Rezivertinib	<50 years: 83.3% (43.6%-97.0%); 50-65 years: 90.0% (69.9%-97.2%); ≥65 years: 76.5% (52.7%-90.4%)
NCT03823807^[18]^	*N*=227 <65 years: *n*=133 ≥65 years: *n*=94	Second or third line	Rilertinib	<65 years: 57.1% (48.3%-65.7%); ≥65 years: 64.9% (54.4%-74.5%)

ORR: objective response rate.

#### 2.1.1 奥希替尼

FLAURA研究^[9]^对比了奥希替尼与第一代EGFR-TKIs吉非替尼或厄洛替尼在*EGFR*突变阳性NSCLC患者中的一线疗效。结果显示，奥希替尼组中位PFS（median PFS, mPFS）显著延长至18.9个月（对照组10.2个月，HR=0.46，*P*<0.001）。尽管该研究未专门针对老年患者，但其亚组分析显示，奥希替尼在老年患者中展现出与年轻人群相当的PFS（<65岁组：HR=0.44，95%CI：0.33-0.58；≥65岁组：HR=0.49，95%CI：0.35-0.67）。在亚洲、中国及日本亚组的试验进一步证实，PFS获益在老年及年轻患者中一致^[19-21]^。

FLAURA研究^[9]^对≥65岁的患者进行了亚组分析，然而，目前奥希替尼对≥75岁患者的安全性和有效性尚不明确。以下几项回顾性研究评估了奥希替尼在该人群中的临床益处。SPIRAL-0研究^[22]^纳入38例≥75岁患者，肺炎发生率达17.5%，显著高于FLAURA总人群的4%及FLAURA日本亚组的12.3%。Yamamoto等^[23]^对132例≥75岁患者的分析显示，中位PFS为19.4个月，1年PFS率为65.8%。与FLAURA数据一致，但≥3级不良事件（adverse events, AEs）发生率更高（达41.7%），且肺炎发生率（17.4%）仍高于FLAURA全球人群的结果。Sakata等^[24]^回顾性研究对比了203例≥75岁与335例<75岁患者，发现两组PFS无显著差异（16.9 *vs* 22.1个月），但老年组因AEs停药率显著升高（28.6% *vs* 14.9%），提示老年患者需加强毒性管理。

二线治疗中，AURA3 III期临床试验^[10]^证实了奥希替尼对T790M突变患者的生存优势：中位PFS达10.1个月（化疗组：4.4个月，HR=0.30，*P*<0.001），≥65岁亚组获益与年轻患者相似。AURA II期扩展研究^[25]^显示，奥希替尼二线治疗的客观缓解率（objective response rate, ORR）为62%，中位PFS为12.3个月，亚组分析同样表明奥希替尼在<65岁与≥65岁的两个人群中获益相似。

专门针对老年患者的研究进一步验证了其适用性。一项纳入≥75岁患者（平均年龄79.9岁）的试验显示ORR为58.3%，与AURA系列研究总人群数据（51%-71%）相当^[26]^。日本一项回顾性研究^[27]^对比了≥75岁与<75岁患者接受奥希替尼二线治疗的疗效差异。疗效方面，两组ORR、PFS及总生存期（overall survival, OS）均无显著差异。安全性方面，该研究并未在≥75岁的患者中观察到更高的AEs发生率。与既往的研究结果不一致的原因可能为，样本量较小，且接受奥希替尼二线治疗的患者可能已经习惯于AEs的管理。

综合现有证据表明，奥希替尼在老年患者中实施的一线及二线治疗方案均能保持与年轻患者相当的临床疗效。但由于老年患者普遍存在肝脏和肾脏代谢及排泄功能下降、免疫系统衰老性改变以及多病共存等生理病理特征，可能导致该群体接受奥希替尼治疗时毒性增加。特别是在≥75岁的患者当中，肺炎的发生率显著上升。提示在该群体中需特别关注肺炎发生的风险及因AEs停药率升高的趋势。

#### 2.1.2 阿美替尼

阿美替尼是我国首个自主研发的第三代EGFR-TKIs，对*EGFR*敏感突变及*EGFR* T790M耐药突变均有较好的抑制效果。由于在APOLLO试验^[11]^中出色的表现，阿美替尼在中国获批上市，用于*EGFR* T790M阳性NSCLC的二线治疗。该研究中总人群ORR达68.9%（95%CI: 62.6%-74.6%），中位OS（median OS, mOS）为30.2个月（95%CI: 24.2-36.4）。在≥65岁的老年患者中，ORR为70.3%（95%CI: 59.8%-79.5%），mOS为28.4个月（95%CI: 22.9-34.7），疗效无显著年龄差异。AENEAS试验^[12]^显示，阿美替尼一线治疗的mPFS较吉非替尼延长9.4个月（19.3 *vs* 9.9个月）。亚组分析表明，无论年龄是否超过65岁，患者死亡风险均显著降低（<65岁：HR=0.44；≥65岁：HR=0.54），提示年龄不影响治疗效果。

现有证据表明，老年患者可以从阿美替尼单药治疗中获得与年轻患者相当的临床益处。但当前研究证据来自于临床试验的事后亚组分析，针对阿美替尼在老年患者（特别是≥75岁高龄患者人群）的前瞻性专项研究仍显匮乏，且缺乏安全性相关的研究。未来需开展针对老年人群的前瞻性多中心研究，重点评估年龄相关的生理改变对阿美替尼疗效持久性以及AEs发生率的影响。

#### 2.1.3 伏美替尼

中国研发的伏美替尼是第一个针对具有经典*EGFR*突变的晚期NSCLC患者mPFS超过20.0个月（20.8个月）的第三代EGFR-TKIs。基于关键III期FURLONG研究^[13]^，该药在中国获批用于晚期NSCLC一线治疗，研究显示，与吉非替尼相比，伏美替尼显著延长mPFS（20.8 *vs* 11.1个月，HR=0.44）。但在亚组分析中，仅<65岁患者显示明确获益（HR=0.36, 95%CI: 0.26-0.51, *P*<0.05），而≥65岁患者HR未达统计学差异（HR=0.68, 95%CI: 0.43-1.09, *P*=0.11）。

Yan等^[28]^回顾性分析了73例接受伏美替尼一线治疗的患者，mPFS为19.5个月（95%CI: 14.6-24.4），低于FURLONG研究^[13]^结果。研究者认为PFS较短可能与试验纳入了更多的男性、脑转移及IV期患者有关。单因素分析显示，PFS与年龄之间没有显著相关性。然而，多因素分析发现，<65岁患者存在延长PFS的潜在趋势（95%CI: 0.10-1.02, *P*=0.053），尽管*P*值仅具有边缘显著性。

ALSC003研究^[29]^证实了伏美替尼对*EGFR* T790M阳性NSCLC患者的疗效显著。<65岁的年轻患者的ORR为72.4%（95%CI: 64.4%-79.5%），≥65岁的老年患者的ORR为77.3%（95%CI: 66.2%-86.2%）。Hu等^[30]^汇总分析了132例中枢神经系统转移患者接受伏美替尼二线治疗的数据，整体中枢神经系统ORR为65%，其中<65岁组为63%，≥65岁组为73%，表明伏美替尼具有跨年龄层的稳定疗效。

虽然在FURLONG研究^[13]^中未能证明伏美替尼能延长≥65岁患者的PFS，但HR值仍提示伏美替尼具有延长老年患者PFS的趋势。多项研究^[28-30]^表明，伏美替尼在老年患者中ORR等关键疗效指标与年轻患者相当，提示其可作为老年患者的有效选择。然而，目前缺乏伏美替尼在老年人群的前瞻性研究，OS获益及安全性数据仍不明确，需进一步开展年龄分层研究以明确长期疗效和不良反应特征。

#### 2.1.4 其他第三代EGFR-TKIs

其他的第三代EGFR-TKIs贝福替尼、瑞齐替尼及瑞厄替尼相继获批，适应证涵盖*EGFR*敏感突变及T790M耐药突变患者的一线与后线治疗。贝福替尼基于NCT03861156研究^[15]^获批用于*EGFR* T790M阳性患者后线治疗，并通过NCT04206072研究^[14]^扩展至一线治疗，但后者亚组分析显示其对≥65岁患者PFS延长未达统计学意义（HR=0.79, 95%CI: 0.43-1.42, *P*=0.43）。基于NCT03812809研究^[16]^，瑞齐替尼获批用于*EGFR* T790M阳性的患者的后线治疗。2022年欧洲肿瘤内科学会（European Society for Medical Oncology, ESMO）公布的IIA期研究^[17]^显示，其一线治疗NSCLC疗效显著，并据此获批用于NSCLC的一线治疗，目前III期临床研究正在进行中。基于NCT03823807研究^[14]^，瑞厄替尼获批用于*EGFR* T790M阳性患者后线治疗。这一系列研究中，除贝福替尼的NCT04206072研究外，其他临床试验均证实第三代EGFR-TKIs在老年与年轻患者中具有相似获益。

尽管初步数据表明第三代EGFR-TKIs在老年患者中疗效优异且耐受性良好，但仍存在显著局限性：首先，除奥希替尼的FLAURA研究^[9]^外，阿美替尼、伏美替尼等药物的关键临床试验（如AENEAS研究^[12]^、FURLONG研究^[13]^）受试者90%以上为中国人群，缺乏高加索人种、非洲裔等群体的药物代谢动力学及疗效数据，这种人群单一性可能导致治疗策略的普适性存疑。其次，针对老年患者这一特殊群体的毒性特征研究尚未系统开展，特别是由肝脏和肾脏的代谢及排泄功能下降、免疫系统衰老性改变以及多病共存等生理病理特征所带来的风险等关键问题仍缺乏循证医学指导。再者，现有回顾性分析的证据等级有限，难以精准指导临床决策。未来需开展大规模前瞻性老年队列研究，为优化该人群的用药策略提供更高级别的循证依据。

### 2.2 第三代EGFR-TKIs联合治疗在老年NSCLC中的研究进展

无论接受何种EGFR-TKIs治疗，最终都会出现获得性耐药。为了延缓耐药、延长生存期、进一步提高疗效，研究人员探索了联合治疗方案，如EGFR-TKIs联合化疗、联合抗血管生成药物或联合双特异性抗体。既往的研究^[31-33]^已经证明，第一代及第二代EGFR-TKIs联合治疗较单用EGFR-TKIs疗效有所提升，第三代EGFR-TKIs联合治疗是否也会带来更好的临床疗效呢?研究人员对第三代EGFR-TKIs联合其他药物的方案进行了探索，表3^[34-37]^汇总了目前第三代EGFR-TKIs联合治疗NSCLC的临床试验及其在老年患者中的疗效。目前联合策略主要聚焦于奥希替尼联合化疗、奥希替尼联合抗血管生成药物、埃万妥单抗联合拉泽替尼。

**表 3:** 第三代EGFR-TKIs联合治疗NSCLC的临床试验

Study	Sample size	Experiment arm	Control arm	Efficacy in all patients	PFS by age HR (95%CI)
FLAURA2^[34]^	*N*=557 <65 years: *n*=340 ≥65 years: *n*=217	Osimertinib+Chemotherapy	Osimertinib	mPFS: 25.5 *vs* 16.7 mon, HR=0.62, 95%CI: 0.49-0.79, *P*<0.001	<65 years: 0.59 (0.44-0.80); ≥65 years: 0.68 (0.47-0.98)
WJOG9717L^[35]^	*N*=122 <75 years: *n*=94 ≥75 years: *n*=28	Osimertinib+Bevacizumab	Osimertinib	mPFS: 22.1 *vs* 20.2 mon, HR=0.86, 95%CI: 0.53-1.40, *P*=0.213	<75 years: 0.76 (0.45-1.34); ≥75 years: 1.11 (0.39-3.16)
RAMOSE^[36]^	*N*=139 <65 years: *n*=65 ≥65 years: *n*=74	Osimertinib+Ramucirumab	Osimertinib	mPFS: 24.8 *vs* 15.6 mon, HR=0.55, 95%CI: 0.32-0.93, *P*=0.023	<65 years: 0.56 (0.26-1.17); ≥65 years: 0.55 (0.26-1.17)
MARIPOSA^[37]^	*N*=858 <65 years: *n*=472 ≥65 years: *n*=386 <75 years: *n*=754 ≥75 years: *n*=104	Amivantamab+Lazertinib	Osimertinib	mPFS: 23.7 *vs* 16.6 mon, HR=0.70, 95%CI: 0.58-0.85, *P*<0.001	<65 years: 0.50 (0.39-0.65); ≥65 years: 1.06 (0.80-1.41); <75 years: 0.70 (0.57-0.85); ≥75 years: 0.77 (0.46-1.30)

mPFS: median PFS.

#### 2.2.1 奥希替尼联合化疗

FLAURA2 III期研究^[34]^显示，针对*EGFR*突变的晚期NSCLC，奥希替尼联合培美曲塞及铂类较单药治疗显著延长PFS（25.5 *vs* 16.7个月；HR=0.62，95%CI：0.49-0.79）。联合治疗中报告了更高的3级以上AEs的发生率（64% *vs* 27%）和更高的血液系统毒性，但这些反应在化疗使用的情况下是预期之中的，并且与单个药物已知的安全性特征一致。同时联合治疗中因AEs导致停药（11% *vs* 6%）、减量（10% *vs* 3%）或剂量中断（43% *vs* 19%）发生率也更高，但剂量中断对奥希替尼的实际暴露总体上影响很小。亚组分析表明，年龄<65岁或≥65岁患者均能从联合治疗中获益（<65岁组：HR=0.59，95%CI：0.44-0.80；≥65岁组：HR=0.68，95%CI：0.47-0.98）。尽管老年患者化疗相关毒性风险需特别关注，该联合方案仍可作为老年患者的有效的治疗选择之一。但临床医生在选择治疗方案时应权衡联合治疗的疗效及安全性，以实现老年患者的个体化治疗。

#### 2.2.2 奥希替尼联合抗血管生成药物

WJOG9717L研究^[35]^评估了奥希替尼联合贝伐珠单抗治疗*EGFR*突变晚期NSCLC的疗效，结果显示联合方案未能显著改善患者PFS（22.1 *vs* 20.2个月，HR=0.86，95%CI：0.53-1.40，*P*=0.213）。安全性方面，联合治疗组3级以上AEs发生率为56%，单药组为48%，表明联合治疗安全性尚可。亚组分析中，<75岁患者PFS有改善趋势（HR=0.76, 95%CI: 0.45-1.34），而≥75岁患者则未见获益（HR=1.11, 95%CI: 0.39-3.16），但差异均无统计学意义。类似结论亦见于8715L^[38]^和BOOSTER^[39]^两项随机临床试验：8715L研究^[38]^显示，对于<75岁或≥75岁患者，奥希替尼联合贝伐珠单抗作为二线治疗均未降低疾病进展或死亡风险，甚至在<75岁的年轻亚组中风险比升高（HR=1.47, 95%CI: 0.78-2.75），BOOSTER研究^[39]^同样证实，<65岁或≥65岁患者均未观察到PFS延长。

OSIRAM-1^[40]^和RAMOSE^[36]^两项研究评估了奥希替尼联合雷莫芦单抗的疗效，但两项研究结果存在矛盾。OSIRAM-1研究^[40]^显示，联合治疗方案相对于奥希替尼单药治疗未展现出显著优势：联合治疗组mPFS为20.0个月，单药组为24.0个月。OS方面，联合治疗组OS为43.3个月，而单药组数据尚未成熟。在≥65岁亚组中同样未观察到显著临床获益。与之形成对比的是RAMOSE研究^[36]^，其结果显示联合治疗组PFS显著优于单药组（24.8 *vs* 15.6个月，HR=0.55，95%CI：0.32-0.93），且≥65岁老年患者亚组PFS延长趋势明显（HR=0.55, 95%CI: 0.26-1.17）。在安全性方面，联合治疗组3级治疗相关AEs发生率为53%（单药组为41%），但未出现新的非预期安全信号。两组间因AEs导致的停药率相似（9.7% *vs* 8.7%），提示联合方案整体耐受性可接受。WJOG9717L^[35]^和OSIRAM-1^[40]^两项研究中，联合治疗在PFS上没有显示出优越性的原因，研究者考虑可能与患者接受抗血管生成药物治疗的持续时间较短有关（8和4.2个月）。

综上，奥希替尼联合抗血管生成药物（贝伐珠单抗/雷莫芦单抗）在延长PFS方面未显现明确优势，未能超越奥希替尼单药或其他第三代EGFR-TKIs的疗效。尽管个别试验提示特定联合方案可能获益，但结果缺乏一致性。在老年患者中，现有研究证据同样未能证明奥希替尼联合抗血管生成药物能显著提升疗效，也未能证明其明确增加了AEs风险。但仍需更多循证医学证据支持其临床应用的价值。

#### 2.2.3 埃万妥单抗联合拉泽替尼

间质表皮转化因子（mesenchymal to epithelial transition factor, *MET*）扩增已被确定为对第三代EGFR-TKIs治疗获得性耐药的主要机制，埃万妥单抗作为同时靶向*EGFR*和*MET*的双特异性抗体，其联合拉泽替尼的疗效在*EGFR*敏感突变患者中进行了探索。MARIPOSA研究^[37]^表明，埃万妥单抗联合拉泽替尼较奥希替尼单药显著降低30%疾病进展或死亡风险（HR=0.70, 95%CI: 0.58-0.85），PFS分别为23.7和16.6个月。但亚组分析显示，当以65岁作为年龄分层的阈值时，65岁以上患者未显示显著获益（HR=1.06, 95%CI: 0.80-1.41），当年龄分层调整为75岁后，联合方案在<75岁和≥75岁亚组均显示疗效优势趋势（<75岁组：HR=0.70，95%CI：0.57-0.85；≥75岁组：HR=0.77，95%CI：0.46-1.30），但是研究组并没有对此现象进行明确解释。在安全性方面，埃万妥单抗联合拉泽替尼组≥3级AEs发生率为75%，奥希替尼组为43%。埃万妥单抗联合拉泽替尼组37%的患者和奥希替尼组9%的患者报告了静脉血栓栓塞AE，联合治疗中因AEs导致剂量中断（83% *vs* 39%）、减量（59% *vs* 5%）或停药（35% *vs* 14%）的发生率也更高。可见，埃万妥单抗联合拉泽替尼组AEs发生率很高，但由于治疗相关AEs导致停用所有药物的情况并不常见，表明大多数患者可以继续接受治疗。

鉴于奥希替尼作为口服药物的便利性和相对可耐受的毒性，为合适的患者探索强化治疗方案变得至关重要。奥希替尼联合抗血管生成药物未显示优势，但奥希替尼联合化疗、埃万妥单抗联合拉泽替尼等联合方案均显示生存延长优势，或可作为老年患者的潜在治疗选择。然而，联合治疗与较高的AEs发生率有关。不同联合治疗方案≥3级AEs发生率见图1^[34-37]^，且不同的联合治疗策略的毒性谱各有特征：联合化疗易导致血液系统毒性增加，联合抗血管治疗可能增加心血管事件发生率。因此治疗方案的选择需系统性评估患者的基础功能、共病状态及老年综合征；并建议采用老年综合评估（Comprehensive Geriatric Assessment, CGA）工具量化营养状况、日常活动能力等预后相关指标，从而为老年患者的精准用药提供高级别证据支持。

**图 1:**
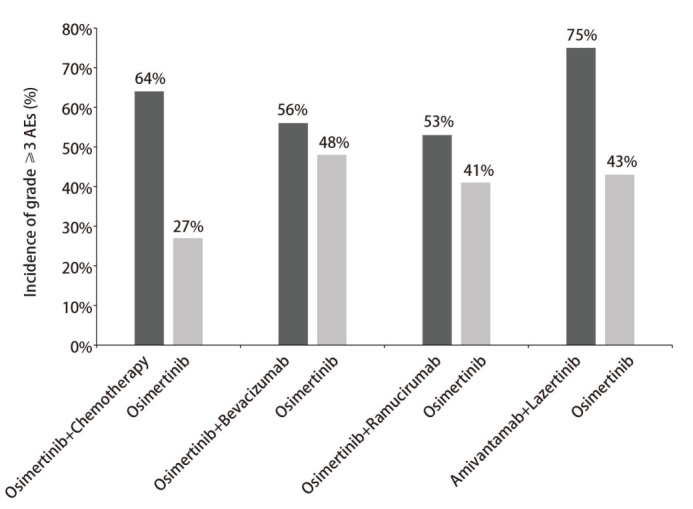
不同联合治疗方案≥3级AEs发生率

## 3 结语及展望

第一代及第二代EGFR-TKIs在老年患者中展现出明确的生存获益，但第三代EGFR-TKIs在老年患者中的疗效尚存争议。本文通过整合前瞻性和回顾性研究中预先指定的目的或亚组分析发现，第三代EGFR-TKIs在≥65岁及≥75岁的患者中展现出与年轻患者相当的PFS和OS，且安全性良好。老年患者特别是≥75岁的患者中，接受奥希替尼时肺炎发生率显著升高，提示在该群体中需特别关注肺炎发生的风险及因AEs停药率升高的趋势。

在联合治疗方面，奥希替尼联合化疗或双特异性抗体等药物虽可显著延长PFS，但AEs发生率同步升高。尽管老年亚组分析显示生存获益持续存在，但需特别关注治疗耐受性下降对临床净获益的影响。

基于现有证据缺口，未来建议开展多中心、跨种族的大规模前瞻性老年队列研究，通过延长随访周期、建立CGA体系、整合循环肿瘤DNA动态监测及药代动力学建模等创新手段，系统解析年龄相关生理改变对药物代谢的影响机制，从而为制定个体化给药方案提供高级证据支持。
